# Comparing Risks of Firearm-Related Death and Injury Among Young Adult Males in Selected US Cities With Wartime Service in Iraq and Afghanistan

**DOI:** 10.1001/jamanetworkopen.2022.48132

**Published:** 2022-12-22

**Authors:** Brandon del Pozo, Alex Knorre, Michael J. Mello, Aaron Chalfin

**Affiliations:** 1 Division of General Internal Medicine, Rhode Island Hospital, Providence; 2 The Warren Alpert Medical School of Brown University, Providence, Rhode Island; 3 Department of Criminology, University of Pennsylvania, Philadelphia; 4 Injury Prevention Center at Rhode Island Hospital, Providence; 5 National Bureau of Economic Research, Cambridge, Massachusetts

## Abstract

**Importance:**

In 2020, homicides in the United States saw a record single-year increase, with firearm injuries becoming the leading cause of death for children, adolescents, and young adults. It is critical to understand the magnitude of this crisis to formulate an effective response.

**Objective:**

To evaluate whether young adult males living in parts of 4 major US cities faced a firearm-related death and injury risk comparable with risks encountered during recent wartime service in Iraq and Afghanistan.

**Design, Setting, and Participants:**

In this cross-sectional study of young adult males aged 18 to 29 years living in the top 10% most violent zip codes in each domestic setting (as measured by fatal shooting rates), fatal and nonfatal shooting data for 2020 and 2021 were aggregated at the zip code level for 4 of the largest US cities (Chicago, Illinois; Los Angeles, California; New York, New York; and Philadelphia, Pennsylvania). Wartime mortality and combat injury rates for the conflicts in Iraq and Afghanistan were used to assess relative risk.

**Main Outcomes and Measures:**

The relative risk of firearm-related death and nonfatal shootings in each setting as compared with combat death and injury in the comparator setting.

**Results:**

Of 129 826 young adult males aged 18 to 29 years living in the top 10% most violent zip codes in the 4 cities studied, 45 725 (35.2%) were Black, 71 005 (54.7%) were Hispanic, and 40 355 (31.1%) were White. Among this population, there were 470 homicides and 1684 firearm-related injuries. Young adult males living in the most violent zip code of Chicago (2585 individuals aged 20-29 y) and Philadelphia (2448 individuals aged 18-29 y) faced a higher risk of firearm-related homicide than US soldiers who were deployed to Afghanistan, with risk ratios of 3.23 (95% CI, 2.47-4.68) and 1.91 (95% CI, 1.32-3.46), respectively. In expanding the analysis to the top 10% of the cities’ most violent zip codes, the risks in Chicago likewise exceeded those of combat death faced by military service members, with a risk ratio of 2.10 (95% CI, 1.82-2.46), and the risks in Philadelphia were comparable with those of deployment to war 1.15 (95% CI, 0.98-1.39). Nonfatal shooting risks were comparable with, or exceeded, the injury risk of combat in Iraq, producing a combined annual firearm risk of 5.8% in Chicago and 3.2% in Philadelphia. However, these findings were not observed in the most violent zip codes of Los Angeles and New York City, where young men faced a 70% to 91% lower risk than soldiers in the Afghanistan war across fatal and nonfatal categories (eg, fatal shooting in most violent zip code in Los Angeles: risk ratio, 0.30; 95% CI, 0.26-0.34; nonfatal shooting in top 10% most violent zip codes in New York: risk ratio, 0.09; 95% CI, 0.08-0.10). The risk of violent death and injury observed in the zip codes studied was almost entirely borne by individuals from minoritized racial and ethnic groups: Black and Hispanic males represented 96.2% of those who were fatally shot (452 individuals) and 97.3% of those who experienced nonfatal injury (1636 individuals) across the 4 settings studied.

**Conclusions and Relevance:**

In this cross-sectional study, for young adult men in several of the communities studied, firearm violence carried morbidity and mortality risks that exceeded those of war. Health equity requires prioritizing effective responses.

## Key Points

**Question:**

How does the risk of firearm-related death and injury for young adult males in parts of 4 major US cities compare with the corresponding risks faced by military personnel deployed to war?

**Findings:**

This cross-sectional study of 129 826 young adult men living in Chicago, Philadelphia, New York City, and Los Angeles in 2020 and 2021 found that young adult males from zip codes with the most violence in Chicago and Philadelphia had a notably higher risk of firearm-related death than US military personnel who served during the wars in Afghanistan and Iraq, while the most violent areas in New York City and Los Angeles were associated with less risk for young adult males than these theaters of war. In all zip codes studied, risks were overwhelmingly borne by young adult males from minoritized racial and ethnic groups.

**Meaning:**

This study’s finding that young adult males of minoritized racial and ethnic groups in parts of major US cities faced greater firearm-related risks at home than did soldiers at war calls for an urgent response that emphasizes violence reduction and trauma-informed interventions.

## Teaser

This cross-sectional study evaluates how the risks of firearm-related death and injury among young adult males living in Chicago, Los Angeles, New York, and Philadelphia compare with those encountered by military service members in Iraq and Afghanistan.

## Introduction

In 2020, US cities experienced a 30% annual increase in homicides,^1^ with firearms becoming the leading cause of death for children, adolescents, and young adults for the first time.^2^ This study used combat mortality and violent injury risks of military service in recent US theaters of war as a benchmark to contextualize this public health crisis and to better frame its health effects on the most exposed communities.

We compare rates of firearm-related homicides and nonfatal shooting injuries among military-age males (ie, young adults aged 18 to 29 years) in selected parts of 4 major US cities with rates of death and injury faced by US soldiers deployed to the recent US wars in Iraq and Afghanistan. Our purpose in doing so is two-fold. First, this analysis provides a basis for comparison between the risks of domestic violent death and injury and those of wartime service, which is commonly regarded as the most extraordinary hazard faced by young adults as an inherent feature of a nation’s civic life. Calibrating domestic risks against this culturally paradigmatic exposure to violence allows us to better characterize fatal violence in domestic settings and prioritize potential responses. Second, the traumas and sequelae associated with encountering death and violence while at war have been well documented and form the bases for support services offered to veterans.^3^ If domestic homicide mortality and injury risks are comparable, it may call for developing similar interventions.

## Methods

This analysis followed the Strengthening the Reporting of Observational Studies in Epidemiology (STROBE) guidelines for comparative cross-sectional studies. Owing to its use of public secondary data and findings reported in the aggregate, in accordance with the Common Rule, this study did not meet the definition of human participant research requiring institutional review board approval or the requirement for informed consent.

The data used for this analysis include all fatal and nonfatal shootings of young adult males recorded as crimes in 2020 and 2021 in Chicago, Illinois; Los Angeles, California; New York, New York; and Philadelphia, Pennsylvania; the 4 largest US cities for which public data on those who were shot were available.^4,5,6,7^ The 2 other cities among the 6 largest in the United States—Houston, Texas, and Phoenix, Arizona—did not have sufficiently detailed publicly available data on the age, race, and sex of individuals killed or injured by firearms to be included. For New York, Chicago, and Philadelphia, we used shooting death and injury data sets made publicly available by each city; for Los Angeles, we extracted firearms death and injury data from a larger public data set of recorded crimes. For all cities, incident-level data included the latitude and longitude of the event and the race, sex, and age or age group (reflecting each city’s particular age reporting intervals) of the individual shot.

Data were aggregated to the zip code level and linked to corresponding demographic characteristics from the US Census Bureau’s American Community Survey 2019 5-year zip code tabulation areas. To determine whether minoritized groups bore a disproportionate burden of firearm violence, each individual’s race was coded as Black, White, or other (American Indian and Alaska Native alone; Asian alone; Pacific Islander alone; some other race alone; and 2 or more races), with Hispanic as an ethnicity. For individual data, the categories were mutually exclusive. For American Community Survey data, race and ethnicity are not mutually exclusive, so Hispanic individuals are also counted among Black and White individuals as well as people of other races. As a result, the data cannot be fully disambiguated by race and ethnicity at the zip code level, and our analysis looked at the collective risk among all young adult males in the communities studied. Zip codes with fewer than 20 000 total residents were excluded to eliminate cases in which a small denominator would yield rates that are overly sensitive to comparably small changes in the prevalence of gun violence. We acquired wartime combat-related mortality and injury counts (including all types of injury resulting from hostile action) for the conflicts in Iraq and Afghanistan from peer-reviewed analyses of US military data.^8,9^ Data covered 2001 to 2014 for the war in Afghanistan and 2003 to 2009 for the war in Iraq, both periods of active combat.

While there is cause to believe the risks faced by soldiers at war vary across time and between units, there is very limited data about the risks of serving in different military units at different times during the Afghanistan and Iraq wars. In an attempt to address this concern, our analysis also considered the mortality and injury data of a single, deidentified Army brigade combat team (BCT) engaged in combat during 15 months of the Iraq War’s so-called surge. Participating in some of the heaviest fighting of the war, it “apparently faced more intense combat than most of the other 19 BCTs that were present during the surge,”^9^ with notably above-average combat death and injury rates.

### Statistical Analysis

All death and injury data were normalized to rates per 100 000 person-years and were used to compute measures of relative risk. Wartime Afghanistan, the more dangerous of the 2 combat theaters, was designated the comparator for risk ratios (RRs). Since death and injury data from all settings were reported as complete populations rather than estimates subject to sampling error, the principal uncertainty in the study’s calculations was the domestic demographic data derived from the US Census Bureau’s random sampling techniques. In acknowledgment of this uncertainty, the bureau provides standard errors that can be used at the zip code level to determine confidence intervals. Our 95% CIs are therefore an expression of the uncertainty of the demographic data in the denominator of our domestic firearms death and injury rate calculations. The code and data used in this analysis are publicly available.^10^ Data analysis was conducted in R version 4.2.0 (R Project for Statistical Computing). We prespecified the level of significance as 2-sided 95% CIs that do not include 1.

## Results

Of 129 826 young adult males aged 18 to 29 years living in the top 10% most violent zip codes in the 4 cities studied, 45 725 (35.2%) were Black, 71 005 (54.7%) were Hispanic, and 40 355 (31.1%) were White. Overall, there were 470 homicides and 1684 firearm-related injuries. Young adult males in Chicago (2585 in the most violent zip code; 12 505 in the top 10% most violent zip codes) and Philadelphia (2448 in the most violent zip code; 12 768 in the top 10% most violent zip codes) faced substantially higher risks of firearm-related homicide and injury than US soldiers who served in one of the nation’s 2 most recent theaters of war (Table 1). In Chicago, this RR was 2.10 (95% CI, 1.82-2.46) for the top 10% most violent zip codes and 3.23 (95% CI, 2.47-4.68) for the most violent zip code; in Philadelphia, it was 1.15 (95% CI, 0.98-1.39) for the top 10% most violent zip codes and 1.91 (95% CI, 1.32-3.46) for the most violent zip code. While the risk of nonfatal shooting injuries was lower in the most violent areas of Chicago (RR, 0.78; 95% CI, 0.68-0.92) and Philadelphia (RR, 0.44; 95% CI, 0.38-0.53) than among US soldiers who served during the Afghanistan war, they were comparable with the risk of being wounded in combat while serving in Iraq (relative risk, 0.70). The RRs for nonfatal shootings in both New York and Los Angeles were similar (0.09; 95% CI, 0.08-0.10 and 0.09; 95% CI, 0.09-0.10, respectively), indicating the risks of firearm injury there were more than 90% lower than among US soldiers who served during the war in Afghanistan. Likewise, fatal shooting risks in these cities were considerably lower than those experienced by soldiers at war (eg, most violent zip code in Los Angeles: RR, 0.30; 95% CI, 0.26-0.34).

**Table 1.** Homicide and Violent Injury Rates in Recent Wars and Selected Zip Codes in 4 US Cities, 2020-2021^a^

Subset	Deaths per 100 000 person-years	Relative risk	Injuries per 100 000 person-years	Relative risk
US combatants of all ages				
Afghan War (comparator)	395	1 [Reference]	3385	1 [Reference]
Iraq War	330	0.84	2380	0.70
1 BCT in Iraq	675	1.71	6990	2.06
**Subset**	**Firearm homicides per 100 000 person-years (95% CI)**	**Risk ratio (95% CI)**	**Firearm injuries per 100 000 person-years (95% CI)**	**Risk ratio (95% CI)**
Chicago, males aged 20-29 y				
Most violent zip code	1277 (975-1849)	3.23 (2.47-4.68)	4487 (3426-6501)	1.33 (1.01-1.92)
Top 10% most violent zip codes	828 (721-972)	2.10 (1.82-2.46)	2639 (2298-3098)	0.78 (0.68-0.92)
Philadelphia, males aged 18-29 y				
Most violent zip code	756 (522-1368)	1.91 (1.32-3.46)	2492 (1721-4511)	0.74 (0.51-1.33)
Top 10% most violent zip codes	454 (388-548)	1.15 (0.98-1.39)	1492 (1274-1799)	0.44 (0.38-0.53)
Los Angeles, males aged 18-29 y				
Most violent zip code	117 (103-135)	0.30 (0.26-0.34)	487 (428-563)	0.14 (0.13-0.17)
Top 10% most violent zip codes	65 (61-68)	0.16 (0.16-0.17)	313 (297-331)	0.09 (0.09-0.10)
New York, males aged 18-24 y				
Most violent zip code	96 (75-135)	0.24 (0.19-0.34)	626 (486-878)	0.18 (0.14-0.26)
Top 10% most violent zip codes	78 (73-84)	0.20 (0.18-0.21)	300 (280-322)	0.09 (0.08-0.10)

Abbreviation: BCT, brigade combat team.

^a^ Age ranges reflect the reporting intervals used by each city. City data reflect firearm homicides and nonfatal shootings. Combat deaths and injuries include all combat-related sources: gunfire, shrapnel, explosions, burns, friendly fire, and others.

Expressed as aggregate probabilities, young men living in Chicago’s most violent ZIP code faced an annual risk of firearm homicide of 1.3% (1277 [95% CI, 975-1849] per 100 000), and a nonfatal shooting risk of 4.5% (4487 [95% CI, 3426-6501] per 100 000). Taken together, they faced an annual probability of firearm violence of approximately 5.8%. In Philadelphia, this probability was approximately 3.2%. In New York City and Los Angeles, the probabilities were considerably lower, at 0.7%, and 0.6%, respectively.

Continuing to use the Afghan War as our comparator, soldiers in the single, heavily engaged Army BCT for which data were available had a relative risk of combat death of 1.71. If considered as an approximation of the upper limit of brigade-level combat mortality risk during recent wars, this risk nonetheless remained lower than the relative risk of firearms homicide experienced by young adult males in the top 10% most violent zip codes in Chicago (RR, 2.10), and the most violent ZIP code in Philadelphia (RR, 1.91). However, young adult males faced a significantly lower risk of nonfatal firearm injury in the most dangerous zip codes of Chicago (RR, 0.78) and Philadelphia (RR, 0.44) than BCT soldiers did of nonfatal combat wounds (relative risk, 2.06).

The risk of firearm death and injury that prevails in the neighborhoods studied here was almost entirely borne by young adult men from minoritized racial and ethnic groups (Table 2). In the Chicago zip codes analyzed, young adult Black males represented 93.9% of the firearm-related homicides (194 of 207) and 94.8% of the nonfatal shooting injuries (626 of 660). In Philadelphia, they were 79.3% (92 of 116) and 76.9% (293 of 381), respectively; Hispanic young adult men accounted for another 12.9% (15) and 17.3% (66), respectively. By comparison, young adult white males were a small percentage of those shot (7.8% [9] and 5.5% [21]). In total, across the 4 cities, in the zip codes most characterized by concentrated levels of firearm violence, young adult Black males accounted for 79.6% of the homicides (374 of 470) and 79.0% of the those nonfatally injured (1330 of 1684); Hispanic men were 16.6% (78) and 18.2% (306), respectively; and young adult White males were 3.8% (18) and 1.8% (30), respectively.

**Table 2.** Total Firearm Homicides and Nonfatal Shootings in Selected Zip Codes in 4 US cities, 2020-2021, With Associated Demographic Burden of Morbidity and Mortality^a^

Subset	Population^b^	Total firearm homicides, No. (%)^c^	Total nonfatal shootings, No. (%)^c^
**Chicago, males aged 20-29 y**			
Most violent zip code			
Overall	2585	66 (100)	232 (100)
Black	2250	62 (93.9)	222 (95.7)
Hispanic	114	2 (3.0)	7 (3.0)
White	193	2 (3.0)	1 (0.4)
Other	142	0	2 (0.9)
Top 10% most violent zip codes			
Overall	12 505	207 (100)	660 (100)
Black	9753	194 (93.7)	626 (94.8)
Hispanic	959	10 (4.8)	26 (3.9)
White	1474	3 (1.4)	4 (0.6)
Other	1278	0	4 (0.6)
**Philadelphia, males aged 18-29 y**			
Most violent zip code			
Overall	2448	37 (100)	122 (100)
Black	2200	36 (97.3)	119 (97.5)
Hispanic	121	0	2 (1.6)
White	122	1 (2.7)	0
Other	126	0	1 (0.8)
Top 10% most violent zip codes			
Overall	12 768	116 (100)	381 (100)
Black	5846	92 (79.3)	293 (76.9)
Hispanic	3552	15 (12.9)	66 (17.3)
White	4651	9 (7.8)	21 (5.5)
Other	2271	0	1 (0.3)
**Los Angeles, males aged 18-29 y**			
Most violent zip code			
Overall	7293	17 (100)	71 (100)
Black	1383	10 (58.8)	53 (74.6)
Hispanic	5899	6 (35.3)	18 (25.4)
White	2266	1 (5.9)	0
Other	3644	0	0
Top 10% most violent zip codes			
Overall	60 351	78 (100)	378 (100)
Black	11 143	41 (52.0)	220 (58.2)
Hispanic	43 901	34 (43.6)	145 (38.4)
White	24 549	3 (3.8)	2 (0.5)
Other	24 659	0	11 (2.9)
**New York, males aged 18-24 y**			
Most violent ZIP code			
Overall	3116	6	39
Black	2381	4 (66.7)	38 (97.4)
Hispanic	654	2 (33.3)	1 (2.6)
White	163	0	0
Other	572	0	0
Top 10% most violent zip codes			
Overall	44 202	69 (100)	265 (100)
Black	18 983	47 (68.1)	191 (72.1)
Hispanic	22 593	19 (27.5)	69 (26.0)
White	9681	3 (4.3)	3 (1.1)
Other	15 538	0	2 (0.8)
**Total across cities**			
Most violent zip code in each city			
Overall	15 442	126 (100)	464 (100)
Black	8214	112 (88.9)	432 (93.1)
Hispanic	6788	10 (7.9)	28 (6.0)
White	2744	4 (3.2)	1 (0.2)
Other	4484	0	3 (0.6)
Top 10% most violent zip codes			
Overall	129 826	470 (100)	1684 (100)
Black	45 725	374 (79.6)	1330 (79.0)
Hispanic	71 005	78 (16.6)	306 (18.2)
White	40 355	18 (3.8)	30 (1.8)
Other	43 746	0	18 (1.1)

^a^ Age ranges reflect the reporting intervals used by each city.

^b^ For American Community Survey zip code population data, Hispanic is recorded as an ethnicity that may include people of any race; Hispanic residents in this column may also appear among Black, White, and other counts. In the zip code population column, Black, White, and other sum to the total, and Hispanic residents constitute an additional count.

^c^ Data provided by each city classified Hispanic individuals as unique, not counted in other categories by race, ie, all counts and percentages by race are considered non-Hispanic, and all counts and percentages, including Hispanic ethnicity, sum to the total number of incidents.

## Discussion

Exposure to combat has been associated with stress-inducing hypervigilance and elevated rates of homelessness, alcohol use, mental illness, and substance use,^11^ which, in turn, are associated with a steep discounting of future rewards.^12^ Our findings—which show that young men in some of the communities we studied were subject to annual firearm homicide and violent injury rates in excess of 3.0% and as high as 5.8%—lend support to the hypothesis that beyond the deaths and injuries of firearm violence, ongoing exposure to these violent events and their risks are a significant contributor to other health problems and risk behaviors in many US communities.^13,14,15^ The findings likewise suggest that urban health strategies should prioritize violence reduction and take a trauma-informed approach at the relevant touchpoints.

The heterogeneity of findings between cities is also worthy of note. Our results show that even in the zip codes with the most violence in New York and Los Angeles, firearm mortality and injury risks were much lower than those in Chicago and Philadelphia, and also much lower than the risks faced in war. Research should continue to determine what factors divide US cities in terms of risk from firearms violence.

### Limitations

This study has limitations. We did not distinguish between the specific assignments a military service member can assume in a combat zone, which can come with varying risks. Nonetheless, the available data suggest that the risks faced by young adult males in the domestic settings analyzed were still greater in many cases than those faced by soldiers who served in one of the most heavily engaged brigades in Iraq. We note that our analysis aggregates risk across zip codes as a proxy for communities without knowing where some individuals resided (as opposed to where they were shot) or distinguishing between lower- and higher-risk young adult male subgroups within these communities. While aggregation across zip codes and theaters of war may provide the bases for a general comparison of risks, it may mask substantially greater mortality risks among the highest risk subsets of young men living in these communities, as well as neglect the elevated risks of service in the most dangerous military roles.

Other factors suggest that this analysis may understate risks of violence and trauma, however. The morbidity and mortality rates presented here are normalized to person-years, while military tours of duty in Iraq and Afghanistan were time-limited exposures of approximately 12 months. By comparison, people are exposed to highly elevated risks of violence each year they live in these zip codes, including by additional, non–firearm-related means not considered in our analysis. In other words, continued exposure to violent death and injury in these communities provides no posttraumatic period for residents,^15^ and the ongoing risks of violence and trauma accumulate over time.

Finally, site selection was driven by the public availability of data and revealed significant heterogeneity between cities. Further research should examine whether the findings extend to large US cities with aggregate homicide rates that are comparable with or greater than those of Chicago or Philadelphia. According to an analysis of recent homicide data, these cities would include New Orleans, Louisiana; Baltimore, Maryland; St Louis, Missouri; Cleveland, Ohio; Detroit, Michigan; Newark, New Jersey; Milwaukee, Wisconsin; Kansas City, Missouri; Memphis, Tennessee; and Cincinnati, Ohio.^16^

## Conclusions

In this cross-sectional study, the population of young adult males in the most violent areas of 2 of the cities studied faced higher mortality risks from firearm-related deaths than US soldiers faced serving in a combat zone during the wars in Afghanistan and Iraq, including in a heavily-engaged frontline unit. This conclusion accords with an older study that characterized the mortality risks in some minoritized US communities as comparable with places the federal government designated as natural-disaster areas, with the corresponding call for a properly resourced response.^17^ Given that the risks of our study were also overwhelmingly shouldered by minoritized racial and ethnic groups, fully delivering on commitments to health equity requires addressing the violence that lies at the root of many behavioral health disparities, in addition to being a health disparity in its own right.

## Notes

Supplement.
Data Sharing Statement
