# Supplementary material for: Comparing Risks of Firearm-Related Death and Injury Among Young Adult Males in Selected US Cities With Wartime Service in Iraq and Afghanistan
Source: JAMA Netw Open. 2022 Dec 22;5(12):e2248132. doi: 10.1001/jamanetworkopen.2022.48132 (PMC9856602; doi:10.1001/jamanetworkopen.2022.48132)
Supplement: Supplement. — Data Sharing Statement [file jamanetwopen-e2248132-s001.pdf]

Data Sharing Statement

del Pozo. Comparing Risks of Firearm-Related Death and Injury Among Young Adult Males in Selected US Cities With Wartime Service in Iraq and Afghanistan. *JAMA Netw Open*. Published December 22, 2022. doi:10.1001/jamanetworkopen.2022.48132

Data
Data available: Yes
Data types: Deidentified participant data
How to access data: https://github.com/alexeyknorre/safer_at_war
When available: With publication

Supporting Documents
Document types: Statistical/analytic code
How to access documents: https://github.com/alexeyknorre/safer_at_war
When available: With publication

Additional Information
Who can access the data: Anyone with the URL.
Types of analyses: For any purpose.
Mechanisms of data availability: Without support; these are already public data.
Any additional restrictions: None. The are already public data.
